# Views and preferences of medical professionals and pregnant women about a novel primary prevention intervention for hypertensive disorders of pregnancy: a qualitative study

**DOI:** 10.1186/s12978-019-0707-8

**Published:** 2019-05-02

**Authors:** A. Vestering, M. N. Bekker, D. E. Grobbee, R. van der Graaf, A. Franx, N. M. T. Crombag, J. L. Browne

**Affiliations:** 1 Julius Global Health, Julius Center for Health Sciences and Primary Care, UMC Utrecht, Utrecht University, Utrecht, the Netherlands; 2 Department of Obstetrics and Gynaecology, Wilhelmina Children’s Hospital, University Medical Centre Utrecht, Utrecht University, Utrecht, the Netherlands; 3 Department of Medical Humanities, Julius Center for Health Sciences and Primary Care, UMC Utrecht, Utrecht University, Utrecht, the Netherlands; 4 Department of development and regeneration, KU Leuven University, Leuven, Belgium

**Keywords:** Hypertensive disorders of pregnancy, Primary prevention, Aspirin, Calcium, Qualitative research, Patient perspective, Women-centred care

## Abstract

**Background:**

Calcium and low-dose aspirin are two potential approaches for primary prevention of hypertensive disorders of pregnancy (HDP). This study aimed to explore the acceptability, views and preferences of pregnant women and primary healthcare providers for a fixed-dose combined preparation of aspirin and calcium (*a polypill*) as primary prevention of HDP in an unselected pregnant population.

**Methods:**

In this qualitative study eight in-depth semi-structured interviews were conducted with Dutch primary care midwives and general practitioners. Seven focus group discussions were organised with women with low-risk pregnancies. Topics discussed were: perceptions of preeclampsia; information provision about preeclampsia and a polypill; views on the polypill concept; preferences and needs regarding implementation of a polypill. Thematic analysis of the data transcripts was carried out to identify emerging themes.

**Results:**

Two major themes shaped medical professionals’ and women’s views on the polypill concept: ‘Informed Choice’ and ‘Medicalisation’. Both could be divided into subthemes related to information provision, personal choice and discussions with regard to the balance between ‘unnecessary medicalisation’ and ‘scientific progress’.

**Conclusions:**

In general, women and healthcare practitioners expressed a positive attitude towards a polypill intervention as primary prevention strategy with aspirin and calcium, providing some conditions are met. The most important conditions for implementation of such a strategy were safety, effectiveness and the possibility to make a well-informed autonomous decision.

**Electronic supplementary material:**

The online version of this article (10.1186/s12978-019-0707-8) contains supplementary material, which is available to authorized users.

## Plain English summary

Why did we do this study? Hypertensive disorders of pregnancy (HDP), such as gestational hypertension and preeclampsia, affect 10–15% of pregnancies in the Netherlands and can lead to complications for both mother and child. Supplementation of calcium and a low dose of aspirin are two potential ways to prevent hypertensive disorders of pregnancy. These can be combined into a single pill, a *polypill*, which could further reduce the risk of HDP. Predicting which women are at risk of HDP has proven to be difficult. Instead, offering all women a polypill as a way to prevent HDP (a public health approach) could be considered.

What did we study? This study aimed to explore the acceptability, views and preferences of pregnant women and primary healthcare providers for the concept of a polypill as a public health intervention for the prevention of HDP.

How did we study this? We performed focus group discussions with healthy pregnant women and interviews with primary care practitioners in the Netherlands.

What did we find? As preeclampsia is considered a serious condition that could harm both mother and child, most medical professionals and pregnant women in this study thought it would be an improvement of care to offer a polypill as a public health intervention, provided that three conditions are met. First, it must be scientifically proven that this polypill is a safe and effective intervention. Second, women should have the ability to make a personal decision about the use of it. Finally, making such a decision is only possible if solid information about HDP and the polypill is provided.

## Background

Hypertensive disorders of pregnancy (HDP) are one of the leading causes of maternal and perinatal death and morbidity [1, 2]. In the Netherlands approximately 10–15% of pregnancies are affected by HDP and the estimated incidence of preeclampsia is 1–2% [3]. Early diagnosis and initiation of appropriate treatment contributes to reducing HDP-associated morbidity and mortality [4]. However, given the potential fulminant course of disease, primary prevention – i.e. preventing HDP from occurring – is key [5].

Based on international literature and previous studies, a number of potential primary prevention interventions have been identified, including low dose aspirin and calcium supplementation [5–13]. These can be combined in a fixed-dose preparation – further referred to as ‘a polypill’– to target several causal risk factors at once and promote adherence [5].

There are two implementation strategies for primary prevention of HDP: based on risk stratification or a public health approach with an unselected population. Supplementation of aspirin and/or calcium have been proven to be safe and effective in women with an increased risk of developing HDP [6, 7, 14, 15]. In the Netherlands use of low-dose aspirin is recommended to high-risk women [16]. Dietary advice regarding calcium intake (minimum of 1000 mg per day) is given to women at moderate risk or higher [17]. However, current risk prediction and stratification performance by history taking remains limited [18–20]. Furthermore, complex risk prediction models with biomarkers and uterine artery Dopplers cannot be readily included in routine antenatal care [20]. As such, a public health approach with an unselected population of pregnant women – i.e. including low risk women – could further reduce the burden of HDP [13, 21].

The strategy to offer a polypill to an unselected population has not been explored yet. This qualitative study was conducted in anticipation of further quantitative assessment of the impact of the polypill concept (i.e. safety, efficacy, cost-effectiveness) and possible implementation of a polypill as a public health intervention in the general pregnant population. It explores the acceptability, views and preferences of (low-risk) pregnant women and primary healthcare providers. This is essential knowledge to meet healthcare-users’ needs and contribute to women-centred care [22–25].

### Methods

In this qualitative study individual in-depth interviews were conducted with health professionals and focus group discussions with pregnant women. All data were collected between July and September 2017.

### Study participants

Within the Dutch perinatal healthcare system a distinction is made between ‘low risk’ care under the supervision of primary care practitioners (midwives or general practitioners) and ‘high risk’ care in hospitals under the responsibility of gynaecologists. As a public health approach primarily includes the general pregnant population classified as ‘low risk’, this study focused on these women and their healthcare providers.

The sample of medical professionals consisted of primary care midwives and general practitioners as potential future prescribers of a polypill, and were selected by purposive sampling [26, 27]. They were invited via email, after which a face-to-face interview was scheduled.

Women who participated in focus groups were also selected by purposive sampling and had to be 18 years or older; with a gestational age of 8 to 24 weeks; a singleton pregnancy; no pregnancy assisted by reproductive technologies; no history of hypertensive disorders in their current or previous pregnancies. Women’s current calcium intake and other additional risk factors for HDP were not included as in-or exclusion criteria. For in a public health approach, the population for whom the polypill is hypothetically intended, is not selected by these risk factors either. Respondents were recruited from community midwifery practices in areas around the cities of Utrecht and Amsterdam. In addition to traditional methods of recruitment, i.e. face-to-face recruitment and distributing flyers and poster, web-based recruitment through an (paid) advertisement on Facebook targeted at the Utrecht province was used to reach a larger and more diverse group of potential participants. Women who expressed their interest in participation, received written information on the study and were scheduled for a focus group.

### Data collection

In-depth interviews were held with midwives and general practitioners using a semi-structured interview protocol. Each interview started with an introduction including information about the polypill concept. Subsequently, the following topics were discussed: perceptions of preeclampsia; information about preeclampsia provided to clients; advantages and disadvantages of the polypill concept; preferences and needs regarding the implementation of a polypill. Interviews lasted approximately 40 min and were audiotaped and transcribed verbatim. When analysis indicated saturation had been reached as no new themes emerged from the data, recruitment was stopped [26]. In qualitative research saturation means the endpoint of data collection [27]. It is important to note that saturation in this qualitative study is reached on themes, not on the number of participants.

Focus group discussions were organised with pregnant women. This methodology was chosen as group dynamics and discussions create a natural environment to explore the different views of pregnant women. It also incorporates that an individual’s attitude and beliefs are socially constructed and people also form their opinions and views by listening and responding to others [28, 29].

The focus groups were conducted using a semi-structured interview protocol. A trained moderator (AV) guided the discussion by asking open-ended questions without participating or sharing personal views. This moderator is not involved in the development of the polypill. Prior to the session participants were asked to complete a questionnaire to collect information on demographics and personal and obstetric history.

Each focus group started with questions about preeclampsia to get a baseline overview of women’s knowledge and perceptions of the target condition. Subsequently, an information video with general information on prevalence and signs and symptoms of HDP was shown to ensure all participants had a similar understanding of the conditions. The video was made in collaboration with the Dutch Society of Obstetrics and Gynaecology and the Royal Dutch Association for Midwives and produced by the Dutch patient organisation, the HELLP Foundation. It is publicly accessible via their website (www.hellp.nl). As the polypill concept is not yet implemented in the Dutch perinatal care system, a hypothetical scenario with explanation about the polypill concept as a public health intervention was presented similarly to the participants to provide focus and promote a more in-depth discussion (see Additional file 1). In this scenario, amongst other polypill characteristics current evidence on safety and effectiveness of calcium and aspirin supplementation was discussed (see Additional file 2). Participants were explained that virtually all studies have been performed in high risk populations. They were informed that as such this evidence is only partly applicable to them.

Subsequently, the following topics were discussed: knowledge and perception of preeclampsia (before and after viewing the information video); perceptions of information about preeclampsia received during pregnancy; advantages and disadvantages of the polypill concept; preferences and needs regarding the use of a polypill. Focus groups lasted approximately 90 min and were audiotaped and transcribed verbatim. When it was agreed among the research group that no new information was collected and thus saturation had been reached [26], data-collection was stopped.

### Data analysis

MAXQDA computer software was used to carry out thematic analysis [27, 30]. To identify emerging themes transcripts were systematically coded using an iterative and inductive process [27]. First, we conducted open coding, in which initial codes were assigned to text fragments. Subsequently, data to data comparison lead to more focused codes. Finally, during axial coding, codes could be categorised and themes and subthemes emerged that related to the broader context of the research subject. The transcripts were coded and analysed by the moderator. Another researcher (NC) analysed samples of the data after which initial and focused coding were compared and reviewed. Subsequently, the analysis was discussed with the research team to form a more representative coding scheme. In consultation with the research team representative quotes were selected and translated by the moderator. As a means of quality control, reversed translation of the quotes was performed by another researcher (JB).

## Results

### Baseline characteristics

Saturation was reached after eight interviews with six midwives and two general practitioners, and seven focus groups with 25 women had been conducted. Tables 1 and 2 show characteristics of medical professionals and pregnant women, respectively.

**Table 1:** Characteristics medical professionals (*n* = 8)

Characteristics	Midwives (*n* = 6)	General practitioners (*n* = 2)
Years of clinical experience (mean, range)	21.3 (2–40)	18
Practice area^a^		
Lower urbanized	4	2
High-urbanized	2	1

^a^The practice area was defined as lower urbanized if the surrounding address density was less than 1500 per km^2^ and high urbanized area if this was more than 2500 addresses per km^2^ [56]

**Table 2:** Socio-demographic characteristics pregnant women (*n* = 25)

Characteristics	
Maternal age (mean, range)	31.4 (24–38)
Parity	(*n* =, %)
Primiparous	18 (72)
Multiparous	7 (28)
Highest level of education	
Vocational	5 (20)
Professional	10 (40)
Academic	8 (32)
Unknown	2(8%)
Living area^a^	
Lower urbanized	11 (44)
High-urbanized	14 (56)
Migration background^b^	
Dutch	23 (9)
non-Dutch	2 (8)
General experience with preeclampsia^c^	
Yes	12 (48)
No	13 (52)

^a^The living area was defined as lower urbanized if the surrounding address density was less than 1500 per km2 and high-urbanised area if this was more than 2500 addresses per km^2^

^b^In the Netherlands migration background is defined by country of birth of a person’s parents. If one or both parents are born outside the Netherlands it is defined as ‘non-Dutch’ [57]

^c^General experience was described as ‘have or have not people in your social environment who have experienced preeclampsia’

### Themes and subthemes

**Fig. 1:**
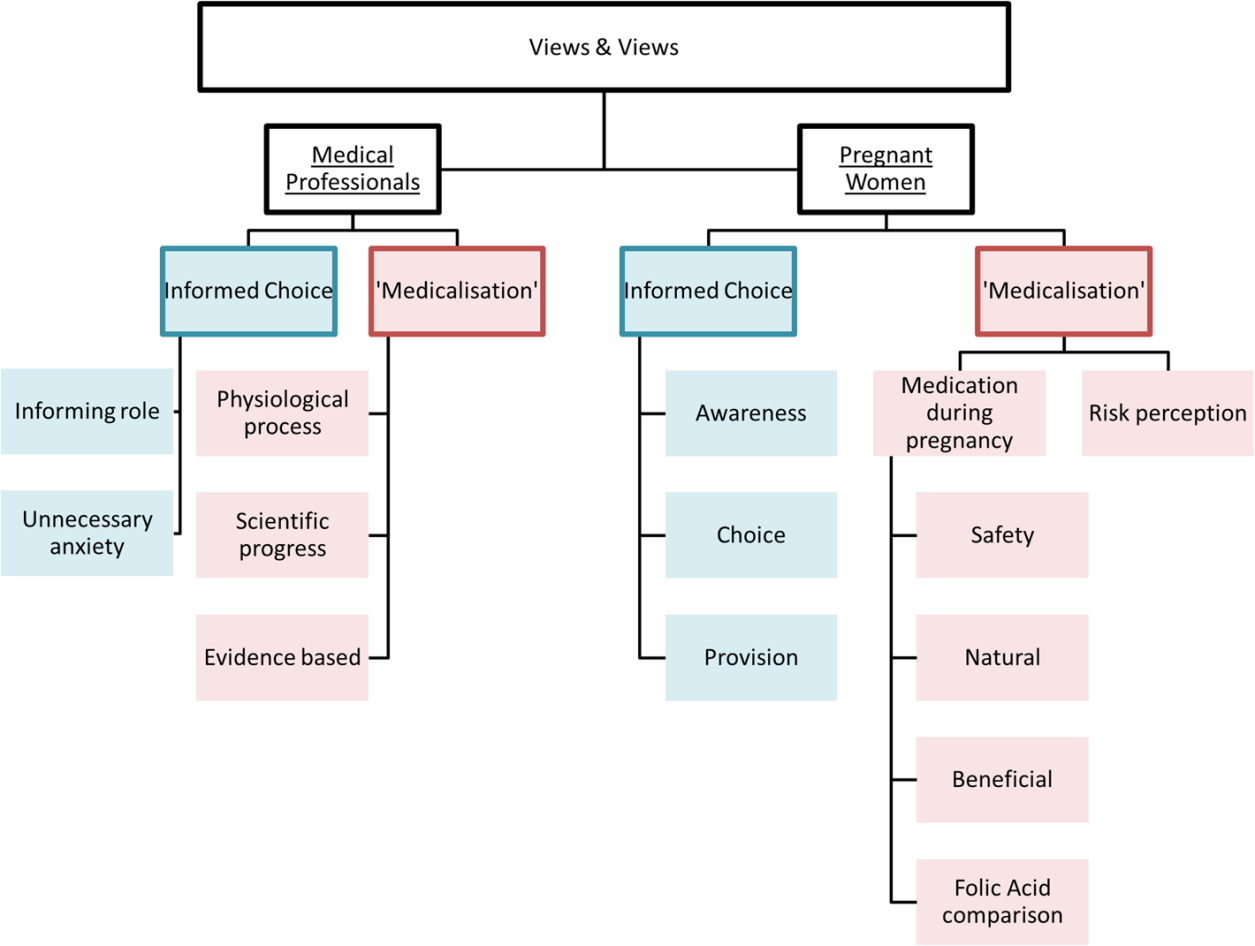
Views and preferences on the polypill: emerging themes and subthemes

We identified two major themes that shaped medical professionals’ and women’s preferences for and views on the polypill concept: ‘Informed Choice’ and ‘Medicalisation’. Each theme could be divided into subthemes for both groups separately (Fig. 1). ‘Informed Choice’ was divided into subthemes related to notions of information provision and personal choice. ‘Medicalisation’ formed the other subthemes and relates to the discussion about the balance between ‘unnecessary medicalisation’ and scientific progress.

### Medical professionals

#### Informing role

All healthcare practitioners emphasised to have an informing role, but that it is ultimately a woman’s own choice to use a polypill or not.

“Our part is to give all the information there is. It isn’t my place to decide for women.” *(PHP1)*

Moreover, if the National Health Council or professional organisations were to include a polypill in national guidelines, all midwives said they would inform women as objectively as possible, irrespective of their personal opinion.

#### Unnecessary anxiety

All medical practitioners agreed that properly informing women is crucial when a new intervention such as a polypill is implemented. This should include information about safety and effectiveness of this polypill and general information about preeclampsia as well. All midwives said that they currently only inform women at increased risk for developing preeclampsia. Some midwives expressed concerns of increased anxiety amongst pregnant women if they provided more information about preeclampsia to low-risk clients. This made them reluctant towards the polypill concept.

“You might alarm people of whom the majority doesn’t have to worry at all. You might make completely normal, uncomplicated pregnancy more stressful.*” (PHP3)*

#### Physiological process vs. scientific progress

One of the major themes during the interviews was potential medicalisation of pregnancy. Most healthcare practitioners said the medical world has to be vigilant not to medicalise pregnancy too much as it is first and foremost a physiological process.

“They keep discovering new things… How far should you go to reduce every single risk in pregnancy?” *(PHP4)*

Other respondents pointed out that scientific progress is part of a medical professional’s job and benefits the ‘patient’ as it could prevent medicalisation later in pregnancy.

“With this pill – this so-called unnecessary medicalisation – you could prevent many interventions you prefer to avoid. I would prefer taking a pill rather than inducing labour, as this could have much more serious complications.” *(PHP6)*

#### Evidence based

Most healthcare practitioners considered ‘significant’ risk reduction in the general pregnant population an important condition for implementation, but safety in particular was considered crucial. Many deemed both aspirin and calcium to be harmless substances as they have been used in obstetric care for many years. One midwife expressed reluctance because of concerns about possible unknown effects on the unborn child.

“We don’t know what harm it could do. No research has been done on a group of healthy pregnant women who took aspirin on a daily basis. You just don’t test medicine on healthy pregnant women.”*(PHP2)*

Whereas some midwives expressed reluctance towards prescribing pills that could have no effect on ‘healthy women’, of whom many would not have gotten ill in the first place, others, however, saw similarities with other ‘harmless’ preventive measures such as folic acid, and saw no disadvantages in using it. Ultimately, most health practitioners thought it would be an improvement of care if a polypill reduces the risk of preeclampsia in the entire population significantly without causing any harm.

“It’s similar to folic acid. For people with a higher risk of having a baby with spina bifida, taking folic acid can prevent this. For others it might not have any effect but it doesn’t cause any harm either.” *(PHP6)*

### Pregnant women

#### Risk perception

Most women had limited knowledge of preeclampsia. Women who knew someone with a history of preeclampsia considered it a serious condition for both mother and child. However, others said they had been unaware of the severity of the complications and incidence so far, but learned about this after watching the information video.

#### Awareness

Some women expressed concerns about the lack of information they had received from their midwives about preeclampsia. Most women preferred to receive more information on symptoms to pay specific attention to. It was perceived to create more alertness rather than unnecessary anxiety. Women expected that this would increase early-recognition, timely consultation of a health professional and potentially prevent complications. Some said that taking a pill on a daily basis would make them more aware too. The possibility of taking a preventive pill and more extensive information on symptoms gave women the sense they could actively take part in managing their pregnancy, which they thought was reassuring.

“It surprises me, I had never seen these figures and the severity of the condition. Considering that, I feel like our midwives haven’t informed us properly.” *(P21)*

#### Choice

All women wished to receive information to personally consider the use of a polypill, including those who expressed preliminary reservations towards taking it. Moreover, it was considered important that everyone, irrespective of one’s risk of preeclampsia, should get the option to make this choice.

“Not giving people a choice is worse than the possibility of worrying them by telling. If you worry about it and there is something available, then at least you can do something about.” *(P11)*

Women preferred information to be provided in a layered fashion as they could choose themselves how much (more) detail they wanted to know.

#### Provision

As the polypill concept is a new intervention most women preferred a medical professional to inform them about it. Their ‘expert opinion’ was considered ‘most trustworthy’ source of information.

“When you go to your midwife, you trust her to be well informed and to know what is best for you. If she advises you to take something you trust her professional judgment.” *(P14)*

In addition to information from medical professionals, women preferred to receive information from ‘renowned’ and ‘trustworthy’ websites. Information had to be unequivocal and was considered to be more reliable if provided by multiple sources.

#### ‘Medication’ during pregnancy

Quotes related to the use of medication during pregnancy are given in Table 3. All women agreed that safety is the most crucial condition for using a polypill. Some participants expressed doubts about the (long-term) safety of – mostly – aspirin, which made them reluctant to use the pill. Others considered both substances to be harmless as they are commonly used. Moreover, most women trusted safety to be ensured before the pill is implemented *(Safety).*

**Table 3:** Pregnant women’s views and preferences regarding the theme ‘Medication during pregnancy’

Safety	“I would want to know more about the side effects and other possible long-term negative effects. That would make the difference for me, if they can say that it is proven to be safe and you don’t have to worry.” *(P02)*
	*“*I still wonder what the side-effects could be. They told me you shouldn’t take any painkillers if you can avoid it. Which makes me wonder, why advice to take as little as possible painkillers and then add to such a pill.” *(P12)*
Natural	“I’d rather not take anything chemical, but if it is proven to be good, I would consider it.” *(P21)*
Beneficial	“It sounds like something that it is relatively common. In this way I can at least try to do something about it. Providing that it doesn’t cause any harm for my child, I wouldn’t mind an extra pill. But it sounds like it only benefits your baby. I think prevention is better than cure.” *(P16)*
Folic acid comparison	“It’s odd, I don’t really understand why I’m having reservations. I mean I took folic acid without thinking… Maybe it’s because it feels a bit experimental still.” (P022) “It has to be advised just like folic acid: whenever you’re pregnant it is best to take folic acid and this polypill. And then that will become general knowledge.” (P03)

Some participants said that they rather avoided taking ‘medication’ in general and especially during pregnancy. In this respect, aspirin ‘felt more like medication’ than calcium, as the latter was considered to be a ‘natural’ substance *(Natural).* However, others felt that there were hardly any disadvantages to the use of the polypill if there were no adverse effects. Under the strict condition of it being scientifically proven to be a harmless method that could benefit their child, they had a positive stance towards the polypill concept *(Beneficial).*

Many women mentioned the similarities between folic acid and the polypill. Yet, in contrast to folic acid, a polypill is still new and fairly unknown, which made some more reluctant to use it. According to the women, emphasis on the resemblance could increase its familiarity and thus increase the credibility and trustworthiness of a polypill *(Folic acid comparison).*

## Discussion

### Main findings

The aim of this study was to explore the acceptability, views and preferences of (low-risk) pregnant women and primary healthcare providers about a polypill with aspirin and calcium as a public health intervention in the general pregnant population to prevent hypertensive disorders of pregnancy. Women and health practitioners expressed a positive, yet cautious attitude towards the polypill concept and identified a number of conditions that should be met to consider it’s use: safety, effectiveness and the possibility to make a well-informed autonomous decision.

### Interpretation

The possibility to make a personal choice based on accurate information was a recurring theme in both groups. This is in line with the increasing emphasis of women-centred care in midwifery care provision in the past decade [31]. Women-centred care involves a midwife-client relationship in which shared decision making is promoted and a woman’s wishes and needs are emphasised [32, 33]. This also involves her need for information [34, 35]. Yet, midwives often underestimate the need for information women have [36], which may result in a discrepancy between women’s need for information and the information they actually receive [36–38]. This was also observed in this study. During the focus groups many women expressed they would have preferred to receive more information about preeclampsia. Yet, all midwives indicated they only provided information on HDP to women at increased risk, some justified this with a fear of increased and unnecessary anxiety amongst pregnant women. If the polypill concept is implemented, this information was considered essential to provide to women, and made health professionals more reluctant towards a public health approach.

The protective gatekeeper role midwives take up a in providing information to women can be explained by their responsibility to minimise both physical and mental harm [38]. Keeping certain information from women is thus an attempt to protect them. As anxiety is common amongst women during pregnancy [39, 40], midwives’ concerns are not completely unfounded. However, both qualitative and quantitative studies demonstrate that women who participated in a first-trimester preventive program for preeclampsia did not report increased levels of anxiety irrespective of their classification as high- or low-risk [41, 42]. Moreover, women in our study expected that more information would create more *alertness* rather than unnecessary *anxiety*. The possibility of taking a preventive pill and receiving additional information on symptoms, gave women the sense they could take part in managing their pregnancy more actively, which they expected to be reassuring. This sense of increased control created by active engagement and information, i.e. agency [43], has been described by others too [43–47], and is in agreement with the general pattern in which people who have an increased sense of control over a health threat, experience less anxiety towards that threat [48]. As such, the individual preferences of women may not be served best when professionals decide for them what is good to know or not to know – as illustrated by our findings and previous studies [36–38]. More importantly, it is precisely this acknowledgement of the individual needs and rights of a patient that is central to ‘patient-centred care’.

Based on safety concerns a similar restrictive, protective gatekeeping role is often taken in the involvement of pregnant women in medical research and pharmaceutical interventions during pregnancy, as is also demonstrated by some of our findings [22, 49–51] This position reveals a precautionary principle attitude, i.e. to avoid an action altogether if the action carries the potential to cause significant harm, even if this is highly unlikely [52]. As adverse effects of medication during pregnancy could harm the mother and foetus, safety concerns are indeed appropriate considerations to balance against the potential benefit [22]. Yet, an overly strict adherence to the precautionary principle results in a ‘precautionary paradox’ [53, 54]: with a categorical exclusion of pregnant women in clinical research because of supposed vulnerability, a potentially harmful lack of scientific evidence on the efficacy and safety of medicines used in pregnancy results [50]. Furthermore, an overly restrictive attitude to avoid highly uncertain adverse effects carries the risk of denying individuals access to interventions that might improve health outcomes [22, 52]. As for example with aspirin and calcium in pregnancy that have been proven to be safe and effective, yet mostly tested in large scale clinical trials with high-risk populations. Indeed, one clinical trial with healthy nulliparous pregnant women was conducted in 1993, in which a greater incidence of abruptio placentae was found amongst the aspirin-treated women [55]. However, these findings have not been confirmed by more recent studies. As such there is clinical equipoise to justify future studies to explore the possible benefits and potential harms of aspirin and calcium supplementation.

Participants in this study echoed the precautionary attitude. Reluctance towards using or prescribing medication during pregnancy, including a polypill, was mostly related to doubts about safety. This notion continued to hold true, even when it was considered that most previous research has shown adverse effects of the use of calcium and aspirin to be highly unlikely. At the same time, participants of both groups in this research stated that being too cautious inhibits progress [22], which is inevitably part of healthcare. While expressing the importance of safety of a polypill and vigilance towards medicalisation, most participants, healthcare practitioners and women, considered a polypill with calcium and aspirin to be a simple, presumably harmless method to decrease the risk of a serious condition.

### Strengths and limitations

The aim of this research was to gain an in-depth understanding of the various views and preferences of pregnant women and primary health providers, and the (qualitative) methodology chosen accordingly. We aimed to include heterogeneous samples for both groups to achieve saturation and improve validity [26]. The participating midwives ranged in clinical experience and worked in different areas. The focus groups comprised women of different educational backgrounds, parity, gestational age and experience with HDP. Although one of the exclusion criteria was a pregnancy assisted by reproductive technologies, we inadvertently included a woman who was under care of a gynaecologist for this reason. As this only became clear on the day of the focus group, she was not excluded as this could have affected the group dynamics of this focus group.

### Recommendations for future research implementation and information provision

**Fig. 2:**
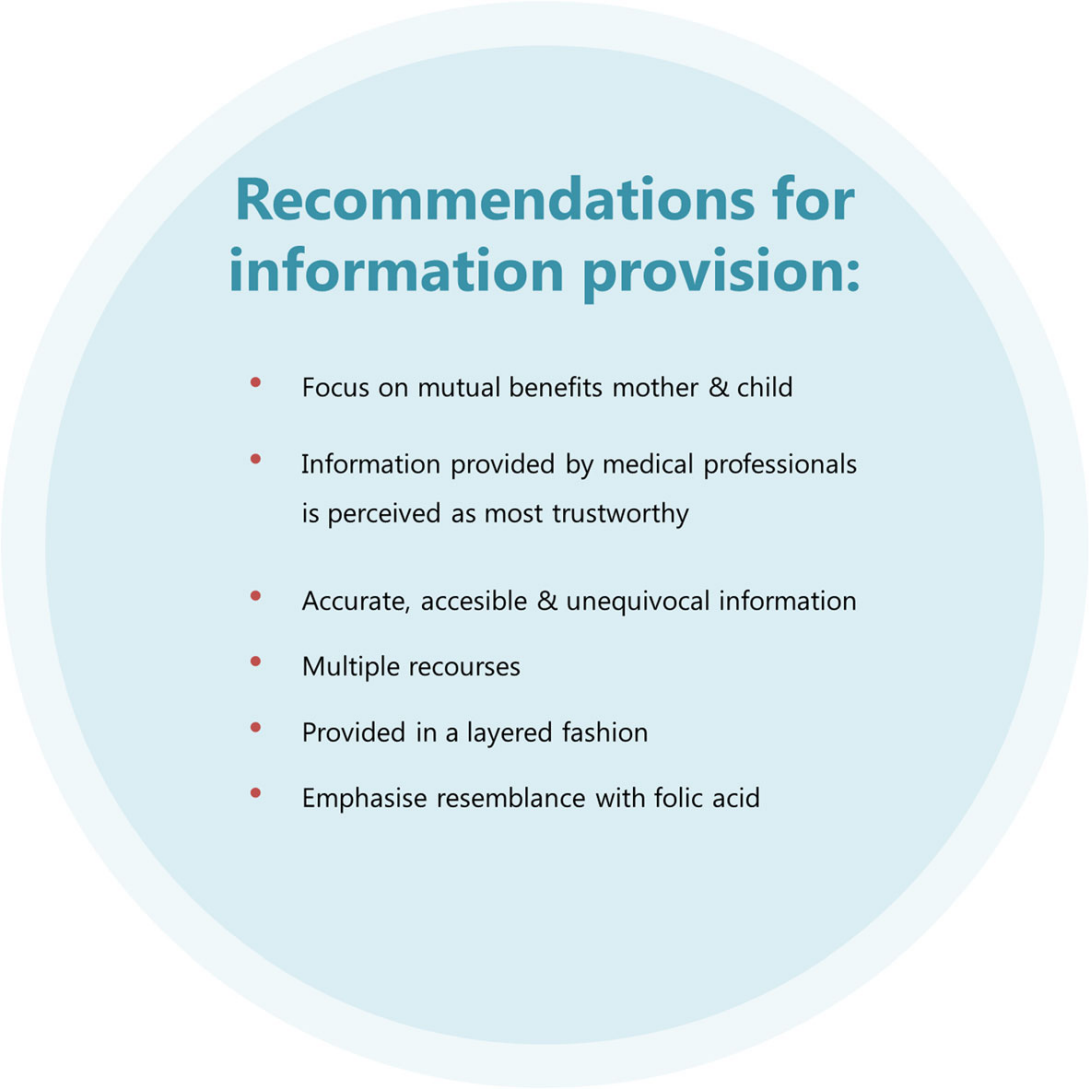
Recommendations for information provision

Applying the results of this research**,** a number of recommendations can be made for future research on the implementation of a public health intervention for an unselected pregnant population. To promote women-centred care, research on safety, effectiveness and implementation strategies is necessary and should include both quantitative assessment (i.e. safety, efficacy, cost-effectiveness) and qualitative evaluation about the opinions and preferences of all stakeholders and implementation enablers and barriers. Recommendations regarding information provision about the polypill are summarised in Fig. 2.

## Conclusion

As preeclampsia is considered a serious condition that could harm both mother and child, most medical professionals and pregnant women in this study thought it had the potential to be an improvement of care to offer a polypill as a public health intervention, provided that three conditions are met. First, more research on effectiveness and safety of this polypill should be conducted. Second, women should have the ability to make an autonomous decision about the use of it. Finally, making such a decision is only possible if solid information about HDP and the polypill is provided.

## Additional files

Additional file 1: Summary of information provision provided in hypothetical scenario polypill. (DOCX 10 kb)

Additional file 2: Results table Browne et al. 2016. (DOCX 13 kb)

## Abbreviation

HDP: Hypertensive disorders of pregnancy

## References

[1] Mol BWJ, Roberts CT, Thangaratinam S, Magee LA, de Groot CJM, Hofmeyr GJ (2016). Pre-eclampsia. Lancet..

[2] Say L, Chou D, Gemmill A, Tunçalp Ö, Moller A-B, Daniels J (2014). Global causes of maternal death: a WHO systematic analysis. Lancet Glob Heal.

[3] Nederlandse Vereniging voor Obstetrie en Gynaecologie (2014). Cardiovasculair risicomanagement na een reproductieve aandoening.

[4] Bhutta ZA, Das JK, Bahl R, Lawn JE, Salam RA, Paul VK (2014). Can available interventions end preventable deaths in mothers, newborn babies, and stillbirths, and at what cost?. Lancet.

[5] Browne JL, Klipstein-Grobusch K, Franx A, Grobbee DE. Prevention of hypertensive disorders of pregnancy: a novel application of the Polypill concept. Curr Cardiol Rep. 2016;18(6). 10.1007/s11886-016-0725-x PMC4875943 27209297

[6] Duley L, Henderson-Smart DJ, Meher S, King JF, Duley L (2007). Antiplatelet agents for preventing pre-eclampsia and its complications. Cochrane Database of Systematic Reviews.

[7] Hofmeyr GJ, Lawrie TA, Atallah ÁN, Torloni MR. Calcium supplementation during pregnancy for preventing hypertensive disorders and related problems. Cochrane Database Syst Rev. 2018;(10):CD001059. 10.1002/14651858.CD001059.pub5 PMC6517256 30277579

[8] Yusuf S, Reddy S, Ounpuu S, Anand S, Sleight P (2002). Two decades of progress in preventing vascular disease. Lancet (London, England).

[9] Lafeber M, Grobbee DE, Schrover IM, Thom S, Webster R, Rodgers A (2015). Comparison of a morning polypill, evening polypill and individual pills on LDL-cholesterol, ambulatory blood pressure and adherence in high-risk patients; a randomized crossover trial. Int J Cardiol.

[10] Wiley B, Fuster V, Shetterly SM, Jackson EA, Keil U (2008). The concept of the polypill in the prevention of cardiovascular disease. Ann Glob Heal.

[11] Webster R, Polypill RA (2015). Progress and challenges to global use---update on the trials and policy implementation. Curr Cardiol Rep.

[12] Wald NJ (2003). Law MR. a strategy to reduce cardiovascular disease by more than 80. BMJ..

[13] Mone F, Mulcahy C, McParland P, McAuliffe FM. Should we recommend universal aspirin for all pregnant women? Am J Obstet Gynecol. 2017;216(2):141.e1–5. 10.1016/j.ajog.2016.09.086 27659212

[14] Souza EV, Torloni MR, Atallah AN, dos SGMS, Kulay L, Sass N (2014). Aspirin plus calcium supplementation to prevent superimposed preeclampsia: a randomized trial. Brazilian J Med.

[15] Rolnik DL, Wright D, Poon LCY, Syngelaki A, O’Gorman N, de Paco Matallana C (2017). ASPRE trial: performance of screening for preterm pre-eclampsia. Ultrasound Obstet Gynecol.

[16] Nederlandse Vereniging voor Obstetrie & Gynaecologie (NVOG) (2018). Wat is de rol van acetylsalicylzuur, gestart ≤16 weken amenorroeduur. ter preventie van pre-eclampsie bij zwangere vrouwen?.

[17] KNOV. Advies over calciumgebruik om zwangerschapsvergiftiging te voorkomen [Internet]. 2016 [cited 2019 Jan 4]. Available from: https://www.knov.nl/actueel-overzicht/nieuws-overzicht/detail/advies-over-calciumgebruik-om-zwangerschapsvergiftiging-te-voorkomen/1798

[18] Poon LC, Nicolaides KH (2014). First-trimester maternal factors and biomarker screening for preeclampsia. Prenat Diagn.

[19] Meads CA, Cnossen JS, Meher S, Juarez-Garcia A, Ter RGDL (2008). Methods of prediction and prevention of pre-eclampsia. Heal Technol Assess.

[20] Lamain-de Ruiter M, Kwee A, Naaktgeboren CA, Louhanepessy RD, De Groot I, Evers IM, et al. External validation of prognostic models for preeclampsia in a Dutch multicenter prospective cohort. Hypertens Pregnancy. 2019;38(2):78–88. 10.1080/10641955.2019.1584210 30892981

[21] Werner EF, Hauspurg AK, Rouse DJ (2015). A cost–benefit analysis of low-dose aspirin prophylaxis for the prevention of preeclampsia in the United States. Obstet Gynecol.

[22] Verweij M, Lambach P, Ortiz JR, Reis A (2017). Maternal immunisation: ethical issues. Lancet Infect Dis.

[23] Fleurence R, Selby JV, Odom-Walker K, Hunt G, Meltzer D, Slutsky JR (2013). How the Patient-Centered Outcomes Research Institute is engaging patients and others in shaping its research agenda. Health Aff.

[24] Wensing M (2003). Improving the quality of health care: methods for incorporating patients’ views in health care. BMJ..

[25] Hibbard JH, Greene J (2013). What the evidence shows about patient activation: better health outcomes and care experiences; fewer data on costs. Health Aff.

[26] Bryman A. Social Research Methods. 4th ed. Oxford: Oxford University Press; 2012. p. 379–414.

[27] Boeije H. Analysis in qualitative research: London: Sage publications; 2009.

[28] Vaughn S, Schumm JS, Sinagub JM. Focus group interviews in education and psychology: Sage; 1996.

[29] Marshall C, Rossman GB. Designing qualitative research: Sage publications; 2014.

[30] VERBI Software. MAXQDA 12, software for qualitative data analysis. Berlin: VERBI Software; 2015.

[31] Stuurgroep Zwangerschap en Geboorte. Een goed begin: veilige zorg rond zwangerschap en geboorte. Adviesrapport aan de minister. 2009;86:25–33.

[32] Nieuwenhuijze MJ, Korstjens I, de Jonge A, de Vries R, Lagro-Janssen A (2014). On speaking terms: a Delphi study on shared decision-making in maternity care. BMC Pregnancy Childbirth.

[33] Epstein RM, Street RL (2007). Epstein 2011, the value of patient-centered care. Patient Educ Couns.

[34] De Boer J, Zeeman KC, Offerhaus P (2008). KNOV-standaard.

[35] Fontein-Kuipers Y, Boele A, Stuij C. Midwives’ perceptions of influences on their behaviour of woman-centered care: a qualitative study. Front Women’s Heal. 2016;1(2).

[36] Proctor S (1998). What determines quality in maternity care? Comparing the perceptions of childbearing women and midwives. Birth..

[37] Scott PA, Taylor A, Valimaki M, Leino-Kilpi H, Dassen T, Gasull M (2003). Autonomy, privacy and informed consent 2: postnatal perspective. Br J Nurs.

[38] Levy V (1999). Protective steering: a grounded theory study of the processes by which midwives facilitate informed choices during pregnancy. J Adv Nurs.

[39] Figueiredo B, Conde A (2011). Anxiety and depression in women and men from early pregnancy to 3-months postpartum. Arch Womens Ment Health.

[40] Huizink AC, Mulder EJH, Robles de Medina PG, Visser GHA, Buitelaar JK (2004). Is pregnancy anxiety a distinctive syndrome?. Early Hum Dev.

[41] Simeone S, Lojo C, Garcia-Esteve L, Triunfo S, Crovetto F, Arranz A, et al. Psychological impact of first-trimester prevention for preeclampsia on anxiety. Prenat Diagn. 2015;35. 10.1002/pd.4485 25156501

[42] Harris JM, Franck L, Green B, Michie S (2014). The psychological impact of providing women with risk information for pre-eclampsia: a qualitative study. Midwifery..

[43] Kornelsen J (2005). Essences and imperatives: an investigation of technology in childbirth. Soc Sci Med.

[44] Langer EJ, Marcus C, Roth J, Hall R (1975). The illusion of control. Soc Psychol (Gott).

[45] Crombag NMTH, Lamain-de Ruiter M, Kwee A, Schielen PCJI, Bensing JM, Visser GHA (2017). Perspectives, preferences and needs regarding early prediction of preeclampsia in Dutch pregnant women: a qualitative study. BMC Pregnancy Childbirth..

[46] Seefat-van Teeffelen A, Nieuwenhuijze M, Korstjens I (2011). Women want proactive psychosocial support from midwives during transition to motherhood: a qualitative study. Midwifery..

[47] Deutsch FM, Ruble DN, Fleming A, Brooks-Gunn J, Stangor C (1988). Information-seeking and maternal self-definition during the transition to motherhood. J Pers Soc Psychol.

[48] Hagger MS, Orbell S (2003). A meta-analytic review of the common-sense model of illness representations. Psychol Health.

[49] Blehar MC, Spong C, Grady C, Goldkind SF, Sahin L, Clayton JA (2013). Enrolling pregnant women: issues in clinical research. Womens Health Issues.

[50] van der Zande ISE, van der Graaf R, Oudijk MA, van Delden JJM (2017). Vulnerability of pregnant women in clinical research. J Med Ethics.

[51] van der Zande ISE, van der Graaf R, Oudijk MA, van Delden JJM (2017). A qualitative study on acceptable levels of risk for pregnant women in clinical research. BMC Med Ethics.

[52] Lewens T (2017). Taking sensible precautions. Lancet..

[53] Sunstein CR (2003). Beyond the precautionary principle. Univ Pa Law Rev.

[54] Tubiana M (2001). The precautionary principle: advantages and risks. J Chir (Paris).

[55] Sibai BM, Caritis SN, Thom E, Klebanoff M, McNellis D, Rocco L (1993). Prevention of preeclampsia with low-dose aspirin in healthy, nulliparous pregnant women. N Engl J Med.

[56] CBS. Begrippen: Stedelijkheid (van een gebied) [Internet]. Den Haag: Centraal Bureau voor de Statistiek (CBS); [cited 2019 Jan 4]. Available from: https://www.cbs.nl/nl-nl/onze-diensten/methoden/begrippen?tab=s#id=stedelijkheid--van-een-gebied--.

[57] CBS. Begrippen: Migratieachtergrond [Internet]. Den Haag: Centraal Bureau voor de Statistiek (CBS); [cited 2019 Jan 4]. Available from: https://www.cbs.nl/nl-nl/onze-diensten/methoden/begrippen?tab=m#id=migratieachtergrond.

