# Supplementary material for: Views and preferences of medical professionals and pregnant women about a novel primary prevention intervention for hypertensive disorders of pregnancy: a qualitative study
Source: Reprod Health. 2019 May 2;16:46. doi: 10.1186/s12978-019-0707-8 (PMC6498498; doi:10.1186/s12978-019-0707-8)
Supplement: Supplementary file 1 — Summary of information provision provided in hypothetical scenario polypill. (DOCX 10 kb) [file 12978_2019_707_MOESM1_ESM.docx]

# Additional file 1

### Summary of information provided in hypothetical scenario polypill

- The Polypill is a combined preparation of:
  - Aspirin (80mg)
  - Calcium (600mg)
- The polypill should be taken on a daily basis from the beginning of the pregnancy until 34 weeks.
- Risk reduction in high-risk population:
  - Aspirin: 10-15%
  - Calcium: 35%
- Both substances are safe to use during pregnancy (tested in high risk pregnancies)
- Advantages / disadvantages of a public health approach for primary prevention of HDP:

| Advantages:   - Possible risk reduction up to 40-50%; - Women who miss out on treatment due to the lack of a robust risk prediction model remain limited. | Disadvantages:   - A large proportion of women who use the pill would never have developed preeclampsia in the first place. |
| --- | --- |
