# Supplementary material for: Views and preferences of medical professionals and pregnant women about a novel primary prevention intervention for hypertensive disorders of pregnancy: a qualitative study
Source: Reprod Health. 2019 May 2;16:46. doi: 10.1186/s12978-019-0707-8 (PMC6498498; doi:10.1186/s12978-019-0707-8)
Supplement: Supplementary file 2 — Results table Browne et al. 2016. (DOCX 13 kb) [file 12978_2019_707_MOESM2_ESM.docx]

# Additional file 2

### Results table Browne et al. 2016

| **Article** | **RCTs included intervention of interest** | **Studies published between** | **Number of participants in all RCTs (in HDP trials)** | **Population*** | **Type of intervention(s)**** | **Start of intervention** | **Control group** | **Estimation of effect PIH** | **Estimation of effect preeclampsia** | **Other subgroup analyses** | **Primary or secondary outcome** | **Side effects reported (in any of the trials)** | **Quality of evidence** | **Included risk of bias assessment** |
| --- | --- | --- | --- | --- | --- | --- | --- | --- | --- | --- | --- | --- | --- | --- |
| Antithrombic therapy, including aspirin | | | | | | | | | | | | | | |
| Duley, 2007 [7] | 59 | 1986-2004 | 37560 (37560) | Heterogeneous | Aspirin (dipyridamole, UFH, LMWH, antithrombin III) |  | Placebo/no treatment, or with another treatment | 34 RCTs, RR 0.95 (0.88-1.03) | 46 RCTs RR 0.83 (0.77-0.89) | High-risk women, PIH (in 12 small RCTs) RR 0.54 (0.41-0.70); PE RR 0.75 (0.66-0.85); | Primary | None | Sufficient | Yes |
| De Jong, 2013 [17] | 9 | 1997-2012 | 1228 (743) | Women with unexplained recurrent miscarriage with or without inherited thrombophilia | Aspirin (UFH, LMWH, nadoparin) | 1st trimester | Placebo/no treatment, or with another treatment | - | None pooled; | Individual studies aspirin or/and LMWH vs no treatment or aspirin-only treatment: not significant | Secondary | Inconsistent reporting of bleeding or local skin reactions with LMHW | Sufficient | Yes |
| Dodd, 2013 [13] | 10 | 1995-2012 | 1139 (761) | Women at risk of placental dysfunction | Heparin, dipyridamole, aspirin, triazolopyrimidine. | Primarily 2nd trimester | No treatment | - | 7 RCTs, RR 0.47 (0.22-1.03) | - | Secondary | Local skin reactions or bruising, in 0.04% placental bleeding with long-term UFH, increased bleeding with regional anesthesia or intraoperative | Sufficient | Yes |
| Askie, 2007 [19] | 31 trials for which individual patient data was available | 1985-2005 | 32217 (32217) | Heterogeneous | Aspirin (dipyridamole, heparin, ozagrel) | 1st and 2nd trimester | Placebo or no treatment | - | RR 0.90 (0.84-0.97) | Similar results restricted to placebo-controlled, aspirin-only, with varying PE definition, or >80% completed follow up trials. No subgroup differences for first or ≥second pregnancy, pre-existing renal disease/ diabetes/hypertension, previous SGA child, maternal age of <20, 20-35 and >35, singleton or multiple, </≥20 weeks of gestation at start, or ≤/> 75 mg aspirin | Primary | No difference in antepartum or postpartum hemorrhage, congenital abnormalities, or increased bleeding infants | High | Yes |
| Bujold, 2010 [20] | 27 | 1985-2005 | 11348 (11348) | Women at increased risk, and started with supplementation </≥ 16 weeks gestation | Aspirin (dipyridamole) | 1st and 2nd trimester | Placebo or no treatment | <16 weeks; 7 RCTs RR 0.62 (0.45-0.84); >16 weeks 14 RCTs RR 0.63 (047-0.85) | <16 weeks; 9 RCTs RR 0.47 (0.34-0.65); >16 weeks 18 RCTs RR 0.81 (0.63-1.03) | No effect of blinding, dose (< or >80 mg), use of dipyridamole, risk of PE (low vs high), or trial size on estimation in sensitivity analysis. | Primary | - | Not assessed except for publication bias | Yes |
| Groeneveld, 2013 [36] | 4 trials for which individual patient data was available | 2004-2009 | 268 (268) | Women with IVF pregnancies, <40 years, varying number of cycles (but <4) | Aspirin | Preconception | Placebo | 4 RCTs, singleton: OR 0.62 (0.22-1.70); twin: OR 1.2 (0.35-4.4) | - |  | Primary | None | Acceptable | Yes |
| Mak, 2010 [21] | 5 | 1996-2009 | 334 (222) | Women with recurrent pregnancy loss and positive anti-phospholipid antibodies | Aspirin + LMWH/UFH | Not reported | Aspirin-only | - | 5 RCTs, RR 0.47 (0.10-2.31) | - | Secondary |  | Low | Yes |
| Diuretics | | | | | | | | | | | | | | |
| Churchill, 2007 [22] | 5 | 1962-1984 | 1836 (1836) | Heterogeneous | Chloorthiazide, hydroclorothiazide, or unspecified thiazide diuretics | 1-3 trimester | Placebo or no treatment | - | 4 RCTs, RR 0.68 (0.45-1.03) | - | Primary | Nausea and vomiting, pruritis and rash, weakness or dizziness | Uncertain | Yes |
| Nitric oxide donors or precursors (L-arginine) | | | | | | | | | | | | | | |
| Meher, 2007 [23] | 6 | 1999-2002 | 310 (310) | Moderate to high risk of developing PE | Glyceryl trinitrate (patches) or oral L-arginine | 1-3 trimester | Placebo/no treatment, or with another treatment | - | 4 RCTs, RR 0.83 (0.49-1.41) |  | Primary | Headache | 4 good quality, 2 uncertain | Yes |
| Dorniak-Wall, 2014 [24] | 7 | 2006-2011 | 884 (228) | Heterogeneous | L-arginine oral or supplement bars | 1-3 trimester | Placebo or not specified for some studies | - | 1 RCT, women at risk for HDP: RR 0.34 (0.21-0.55) | 1 RCT, progression to PE for women with HDP: RR 0.21 (0.05-0.98) | Primary | 1 study reported increase in dyspepsia, nausea, dizziness, headache and palpations | Fair | Yes |
| Metformin | | | | | | | | | | | | | | |
| Feng, 2015 [25] | 5 | 2004-2011 | 932 (673) | Women with polycystic ovary syndrome (PCOS) | Metformin | Pre-conception, or 1st trimester | Stopped metformin after confirmed pregnancy |  | 3 RCTs, RR 0.92 (0.28-3.00) |  | -Primary | Vomiting, diarrhea, gastrointestinal symptoms | Unclear | No |
| Progesterone | | | | | | | | | | | | | | |
| Wahabi, 2001 [26] | 4 | 1987-2009 | 421 (337) | Women with threatened miscarriage | Dydrogesterone, progesterone | 1st trimester | Placebo or no treatment | 2 RCTs, RR 1.00 (0.54-1.88) | - | - | Primary | None | Low | Yes |
| Calcium | | | | | | | | | | | | | | |
| Hofmeyer, 2014 | 13 | 1990-2007 | 15730 (15730) | Heterogeneous | 1.5-2 grams calcium | 1-2 trimester | Placebo or no treatment | 12 RCTs RR 0.65 (0.53-0.81) | 13 RCTs, RR 0.45 (0.31-0.65) | *Low calcium diet: 8 RCTs RR 0.36 (0.20-0.65); high risk of PE: 5 RCTs RR 0.22 (0.12-0.42) | Primary | Difficulty swallowing or chewing, HELLP syndrome (2 trials), reduced bone density with abrupt discontinuation postpartum (1 trial), doses >800mg may reduce iron absorption (1 trial) | High | Yes |
| Hofmeyer, 2014 | 10 | 1987-2006 | 2234 (2234) | Heterogeneous | <1 gr calcium (linoleic acid or antioxidants) | 1-2 trimester | Placebo or no treatment | 5 RCTs, RR 0.53 (0.38-0.74) | 10 RCTs RR 0.38 (0.28-0.52) | *High quality: 1 RCT 0.30 (0.06-1.38); calcium-only: RR 0.36 (23-0.57; calcium plus linoleic acid RR 0.23 (0.09-0.60); calcium plus vitamin D RR 0.49 (0.06-1.01). | Primary |  | Low | Yes |
| Imdad, 2011 [27] | 10 | 1989-2009 | 11405 (11405) | Women in developing countries; primiparous and multiparous, singleton and multiple gestation | Calcium | 1-3 trimester | Placebo | 6 RCTs, RR 0.55 (0.36-0.85) | 10 RCTs, RR 0.41 (0.24-0.69) | Risk PE with higher pre-pregnancy risk: RR 0.18 (0.07-0.42) | Primary | Not assessed | Moderate to high | Yes |
| Vitamin B6 | | | | | | | | | | | | | | |
| Salam, 2015 [28] | 4 | 1963-1984 | 1646 (1197) | Heterogeneous | Pyridoxine-HCL, pyridoxine/multivitamin | 1-3 trimester | Placebo or no treatment, multivitamine without B6 | - | 2 RCTs tablet, RR 1.71 (0.85-3.45), 1 RCT lozenges, RR 1.43 (0.64-3.22) |  | Primary | None | Low | Yes |
| Vitamin C | | | | | | | | | | | | | | |
| Rumbold, 2015 [29] | 29 | 1979-2014 | 24300 (21956) | Heterogeneous | Vitamine C, combined with vitamin E, allopurinol, aspirin, fish oil, or iron/folic acic/vitamin B. | 1-3 trimester | Placebo or no treatment, multivitamins without vitamin C. | - | 16 RCTs, RR 0.92 (0.80-1.05) |  | Primary | None | Moderate quality | Yes |
| Vitamin D | | | | | | | | | | | | | | |
| De-Regil, 2012 [30] | 6 | 1980-2008 | 1023 (400) | Heterogeneous | Vitamin D | 2-3 trimester | Placebo or no treatment | - | 1 RCT, RR 0.67 (0.33-1.35) | - | Primary | None | Very low | Yes |
| Pérez-López, 2014 [31] | 13 | 1980-2014 | 2299 (654) | Heterogeneous | Vitamin D2 or D3 (with calcium, iron or mutlivitamin) | Not reported | Placebo or no intervention |  | 3 RCTs, RR 0.88 (0.51–1.52) |  | Primary | None | Moderate quality | Yes |
| Vitamin E | | | | | | | | | | | | | | |
| Rumbold, 2015 [37] | 21 | 1996-2014 | 22129 (20878) | Heterogeneous | Vitamin E alone, vitamin E/C (with allopurinol, aspirin, fish oil). | 1-3 trimester | Placebo or no treatment. |  | 14 RCTs, RR 0.91 (0.79-1.06) | No difference for <20 or >20 weeks gestation, low or adequate dietary vitamin E intake, low or high risk of adverse pregnancy outcomes at enrolment | Primary | Self-reported abdominal pain, PROM | Moderate quality | Yes |
| Fatty acids supplementation | | | | | | | | | | | | | | |
| Makrides, 2006 [32] | 6 | 1992-2003 | 2783 | Heterogeneous | DHA, EPA, (primrose oils with fish oil) | 2-3 trimester | Placebo or no intervention | 5 RCTs, RR 1.09 (0.90-1.33) | 4 RCTs, RR 0.86 (0.59-1.27) | No differences in timing of supplementation, exclusively marine oils, or high risk | Primary | None | Three low risk. |  |
| Allen, 2014 [33] | 17 | 1992-2012 | 8712 (4579) | Heterogeneous | DHA, EPA, GLA; Omega 3 | 2-3 trimester | Placebo or no intervention | - | 6 RCTs, RR 0.92 (0.71-1.18) |  | Primary | None | Not specified for this interventions | Yes |
| Magnesium | | | | | | | | | | | | | | |
| Makrides, 2014 [34] | 10 | 1979-2007 | 9090 (1042) | Primiparous and multiparous, single and twin gestations, low risk and high risk pregnancies | Magnesium oxide, magnesium citrate, magnesium, magnesium gluconate, magnesium aspartate | 1-3 trimester | Placebo or no treatment | 3 RCTs, RR 0.39 (0.11-1.41) | 3 RCTs, RR 0.87 (0.58-1.32) | No difference for type of magnesium | Primary | Gastro-intestinal symptoms (not significant) | Most trials unclear to high risk, 2 low risk | Yes |
| Zinc | | | | | | | | | | | | | | |
| Ota, 2015 [35] | 21 | 1983-2014 | >17000 (2975) | Primiparous and multiparous, single and twin gestations, low and unreported zinc status | Zinc | 1-3 trimester | Placebo, iron/folic acid | 7 RCTs, RR 0.83 (0.64-1.08) | - |  | Secondary | None | Risk unclear in half study | Yes |
| Garlic | | | | | | | | | | | | | | |
| Meher, 2006 [38] | 1 | 2001 | 100 (100) | Primigravida, moderate risk (positive roll-over test) | Garlic tablets | 2-3 trimester | Placebo | 1 RCT, RR 0.50 (0.25-1.00) | 1 RCT, RR 0.78 (0.31-1.93) | - | Primary | Odour | Uncertain | Yes |
| UFH unfractioned heparin, LMWH low molecular weight heparin, DHA docosahexaenoic acid, EPA eicospentaenoic acid, GLA gammalinoleic acid  Heterogeneous: no consistent inclusion criteria based on risk, parity, singleton or twin pregnancy, age, etc. | | | | | | | | | | | | | | |
